# New Styptic

**Published:** 1853-07

**Authors:** 


### *New Styptic.*

This is a new article for arresting hemorrhage, and is made as follows: "Gum Benzoin, eight ounces; alum, a pound; water ten pints. The alum and Benzoin are boiled for eight hours in the water, fresh water being added to make up for loss by boiling. "The supernatant liquor is the styptic in question," one drop of which should coagulate a basin of blood in an instant. This was introduced by Signor Pagliare of Rome. A Mr. Richardson has been testing this agent, and reports as follows:

"In a patient belonging to Dr. Willis, of Barnes, in whom profuse hemorrhage was taking place from the rectum, the styptic solution was used as an injection, at the suggestion of the speaker. The hemorrhage was effectually checked. It was right, however, to state that the patient was at the same time taking gallic acid in liberal doses. Lastly, he (Mr. Richardson) had reeently had an excellent opportunity of testing the effects of this new styptic in comparison with old ones. Six leeches had been applied to the chest of a child under his care, and the friends had summoned him in great haste to check the bleeding, which was most profuse. There were three points from which the blood flowed; to one of these points the new styptic was freely applied, to another a saturated solution of alum, and the third was touched over with lunar caustic. In all three instances the styptics were only partially successful. The caustic, however, was most efficient, the alum solution next so, and Pagliare's solution least of all. They were all tried as long as was compatible with safety, and, finally, pressure was used with perfect success. Mr. Richardson's opinion, therefore, was, that the styptic powers of the solution of Signor Pagliare had been much overrated. That it was a styptic he had no doubt, for it contained alum. He did not know whether in the mixture a chemical combination was formed betwixt the alum and the benzoic acid; but he thought not. It was, probably, a mere solution of alum containing benzoic acid."

